# Against pragmatism: on efficacy, effectiveness and the real world

**DOI:** 10.1186/1745-6215-10-48

**Published:** 2009-07-06

**Authors:** David M Kent, Georgios Kitsios

**Affiliations:** 1 Institute for Clinical Research and Health Policy Studies and Department of Medicine, Tufts Medical Center, Tufts University School of Medicine, Boston, USA

## Abstract

Explanatory and pragmatic trials represent ends of a continuum of attitudes about clinical trial design. Recent literature argues that pragmatic trials are more informative about clinical care in the real world. Although there is place for more pragmatic studies to inform clinical practice and health policy decision-making, we are concerned that it is generally under-appreciated that extrapolating the results of broadly inclusive pragmatic trials to the care of real patients may often be as problematic as extrapolating the results of narrowly focused explanatory or efficacy trials. Simplistic interpretation of pragmatic trials runs the risk of driving harmful policies.

## Background

Determining the 'true' treatment effect of a given therapy is a bit like determining the 'true' weight of a liter of water. Those who answer that a liter of water weighs a kilogram are either assuming an implicit 'on planet earth, at sea level, at four degrees Celsius' or confusing the intrinsic property of mass with the extrinsic property of weight. Like weight, treatment effect is an extrinsic property, emerging only through an interaction between the intervention, the patient, and the circumstances in which it is being measured [1]. Adjust the context and a different effect emerges – just as a liter of water weighs a little over a third of a kilogram on Mars.

Treweek and Zwarenstein present a narrative review on pragmatic trials highlighting the importance of considering the context of a trial when interpreting its result [2]. Efficacy (or 'explanatory') trials, they argue, with their emphasis on ideal patients in ideal settings, may not generalize to routine care and are therefore not especially informative regarding practical clinical and policy decisions. What is needed, they assert, is a more pragmatic attitude toward trial design, such that trials include all patients who might be considered for a therapy in the real world, so that the trial result can be applicable to practical decisions. Framed in this way, who can argue that trials should not be more pragmatic? Indeed, the argument has been gaining momentum, and we see a welcome shift to a more pragmatic attitude [3,4]. Yet, on the cusp of the era of comparative effectiveness, it is essential that we examine critically the ability of pragmatic trials to make up for this well-appreciated limitation of efficacy trials and provide information directly applicable to the real world.

## Discussion

First, let us recognize that if the purpose of a trial is to examine whether a treatment for a given condition works at all or not (perhaps the most common reason for a trial), then designing a trial toward the explanatory/efficacy end of the continuum is probably a wise decision. The US Food and Drug Administration guidance on this gets to the heart of the issue – a trial may yield null results for a myriad of reasons [5]: the therapy may just not work, or the therapy may work in more carefully diagnosed patients, or those better selected in terms of disease severity, or in those with less co-morbidities or better compliance, or with closer monitoring, or in settings with a higher commitment to and/or more experience with the intervention. The therapy may even work in the enrolled patients in the trial setting, but measurement error obscured the treatment signal or inadequate sample size drowned it in statistical noise. Thus, a null pragmatic trial provides little information about whether our treatment has some potential value. On the other hand, a null efficacy trial, performed under the most favorable possible circumstances, can give us definitive information that a therapy is not of value.

Treweek and Zwarenstein concede that proof of efficacy may be an important pre-requisite for an effectiveness trial [2]. The question then becomes how does one interpret the results of a pragmatic trial in the context of a previously favorable efficacy trial? If only pragmatic trials have implications for 'real world' settings, as the authors suggest, then the results of these trials presumably trump or nullify the results of efficacy trials. Thus, if a broadly inclusive effectiveness trial yields null results, policy decisions should reflect the fact that the treatment does not work in the real world, even when efficacy was demonstrated in a more 'explanatory' trial. Another view, the view that we hold, is that when effectiveness and efficacy trials yield discordant results, the therapy works in some situations and not in others. Physician judgment is then necessary to make treatment decisions based on the degree to which specific patients and settings in the real world correspond to those in the efficacy trial. Indeed, this is not so terribly different from what we might have recommended before the pragmatic trial; the negative effectiveness trial provides important information about how cautious physicians need to be in generalizing the efficacy results.

So, if null efficacy trials are more informative than null effectiveness trials, surely positive pragmatic effectiveness trials must be more informative than positive efficacy trials? However, consider also the potential of positive pragmatic trials to elevate low-value or even harmful care to the status of evidence-based medicine. A broadly inclusive trial is likely to include many patient groups where there is little likelihood of any benefit. For example, we would not be at all persuaded to add a new oral hypoglycemic to the standard regimen in an 85-year-old woman with recent onset diabetes, Class II heart failure and a glycated hemoglobin of 8, who is on five other medications, even if the patient meets the inclusion criteria of a well-conducted effectiveness trial which showed overall benefit – simply because the potential for benefit is too small to warrant the risks of polypharmacy. Similarly, for any treatment with a narrow therapeutic index, we would withhold therapy from patients highly likely to be poorly adherent, even if such patients were included in a positive pragmatic trial.

The issues of extrapolating trial results to patients are still more complex than in these relatively straightforward examples, since heterogeneity of baseline risk can result in situations in which the average result of a trial may be misleading even about the typical patient in the trial itself [6,7]. Unless accompanied by more careful analytic approaches [7-10], broadly inclusive pragmatic trials (which increase the baseline heterogeneity of outcome risk, competing risk, and risk of treatment-related harm) have the potential to exacerbate, rather than reduce, the difficulty of applying clinical trial results to individual patients [11].

The last point we wish to make concerns the increased attention with which care is delivered in the context of a trial. Finding a signal of efficacy can be difficult and additional resources are often used to ensure that performance of therapeutic procedures or adherence to a medical regimen is especially meticulous. Unfortunately, helping patients in the real world is also often difficult and frequently requires meticulous care, and some new therapies may require substantial organizational or structural changes to be effective. While ultra-meticulous care in trials can give misleading results (that are potentially harmful to patients if generalized to inappropriate settings), 'pragmatically' testing such new therapies in resource-challenged environments may often be a recipe – or an excuse – for inertia; it sets so-called 'usual care', rather than the 'best attainable and sustainable' care [12], as the aspirational standard.

## Conclusion

This is a very partial account of the limitations of pragmatic trials. More issues will undoubtedly be revealed as the pendulum swings in the pragmatic direction and we gain more experience with this type of trial. The main point we wish to emphasize is that while both types of trials yield useful information, pragmatic trials do not provide a more accurate measure of the 'true' treatment effect, since the concept of a true effect is fundamentally illusory. While extrapolating the results of efficacy trials to the care of individual patients in the real world can be problematic, and requires careful physician judgment and decision-making, the same is unfortunately true for the results of effectiveness trials. Unless more attention is paid to these under-appreciated limitations, pragmatic trials run the risk of driving harmful policies.

## Competing interests

DMK was partially supported by a grant from the NIH (NIH/NCRR 1UL1 RR025752). GK is Pfizer-Tufts Medical Center Research Fellow in Clinical Research.

## Authors' contributions

DMK and GK discussed the concepts in the manuscript. DMK wrote the first draft. Both authors revised the manuscript for important content and approved the final manuscript.
